# Problem Gambling and its Relation to Delayed Reward Discounting: A Secondary Analysis of a Randomized Controlled Trial

**DOI:** 10.1007/s10899-025-10458-z

**Published:** 2025-11-12

**Authors:** Dominic Haag, Andreas Meyer, Nikolaos Boumparis, Andreas Wenger, Christian Baumgartner, Doris Malischnig, Matthew T. Keough, David C. Hodgins, Yasser Khazaal, Severin Haug, Michael P. Schaub

**Affiliations:** 1 Swiss Research Institute for Public Health and Addiction, University of Zurich, Konradstrasse 32, 8005 Zurich, Switzerland; 2 Department of Adult Psychiatry and Psychotherapy, Psychiatric University Clinic Zurich and University of Zurich, Zurich, Switzerland; 3 Therapy Center Ybbs, Vienna Healthcare Group, Ybbs an der Donau, Austria; 4 Department of Psychology, York University, Toronto, ON Canada; 5 University of Calgary, Calgary, Canada; 6 Department of Addiction Medicine, Faculty of Biology and Medicine, University of Lausanne, Lausanne, Switzerland

**Keywords:** Problem gambling, Delayed reward discounting, Temporal discounting, impulsivity, adherence, Randomized controlled trial

## Abstract

Delayed reward discounting (DRD) refers to the phenomenon where future rewards are perceived as less valuable compared to immediate rewards. The extent of this devaluation has been repeatedly linked to addictive behaviors. DRD could play an important role in the development and maintenance of problem gambling. We aim to better understand the relationship between DRD and problem gambling, examine temporal changes in DRD, and evaluate its predictive value for adherence. We conducted a secondary analysis of data from participants in the Win Back Control study (*n* = 345), a two-arm RCT that demonstrated effectiveness in reducing problematic gambling behavior. DRD was assessed using the Monetary Choice Questionnaire (MCQ), the severity of problem gambling was measured with the Problem Gambling Severity Index (PGSI), and gambling symptom severity was evaluated using the Gambling Symptom Assessment Scale (G-SAS). DRD is significantly positively associated with gambling symptom severity (G-SAS: b = 13.90, *p* = 0.002) and problem gambling severity (PGSI: b = 9.39, *p* < 0.001). DRD decreased significantly on a weekly basis over the three measurement time points (b = -0.001, *p* < 0.001). DRD could not be identified as a mediator, which should be further investigated in future studies. DRD is significantly positively associated with problem gambling severity and gambling symptom severity. DRD does not serve as a predictor of adherence. DRD decreases over the three time points along with severities of gambling behavior and gambling symptoms. However, the findings are correlational and may be affected by attrition bias.

## Introduction

Due to its substantial individual and social costs, problem gambling is considered a significant public health concern (Lancet, 2017; Wardle et al., 2019). Problem gambling not only affects the individual but also places a considerable strain on the social and family environment, potentially leading to the disintegration of family structures (Dowling et al., 2014; Muelleman et al., 2002; Suomi et al., 2022). According to Delfabbro (2013), the term problem gambling is commonly used in two distinct ways. On the one hand, it refers to a less severe form of gambling disorder, which is largely defined in terms of the mechanisms central to substance disorders, such as cravings, tolerance and withdrawal. In this understanding, problem gambling refers to risky or excessive gambling behavior that can lead to negative consequences but does not necessarily meet the diagnostic criteria for a clinical disorder. A diagnosis according to the Diagnostic and Statistical Manual of Mental Disorders V (DSM-V) requires at least four criteria within a 12-month period, characterizing pathological gambling as persistent and recurrent gambling behavior that leads to significant impairment or distress (American Psychiatric Association, 2022). On the other hand, the term problem gambling is also used within a public health framework, where it is conceptualized primarily in terms of its harmful consequences (Delfabbro, 2013). In this study, problem gambling is determined using the Problem Gambling Severity Index (PGSI) (Ferris et al., 2001), a measurement instrument whose items refer to the past 12 months and are closely aligned with the criteria of the DSM-IV (American Psychiatric Association, 1994). Accordingly, the PGSI includes only two items that assess the problematic consequences of gambling (Delfabbro, 2013; Ferris et al., 2001). Thus, in this study, problem gambling is understood in the first sense described above, aligning with the perspective of Jazaeri and Habil (2012), who argue that if a problem gambler meets certain criteria, severe problem gambling may be diagnosed as pathological gambling. The global standardized prevalence rate of problem gambling is estimated to be approximately 2.3%. In general, the lowest standardized prevalence rates of problem gambling tend to occur in Europe, with intermediate rates in North America and Australia, and the highest rates in Asia (Williams, 2012).

Delayed reward discointing (DRD) constitutes a behavioral economic construct that reflects the decrease in the present value of an outcome as its receipt is delayed (Mazur et al., 1987). The rate at which delayed rewards lose their subjective value can be empirically assessed through various hypothetical or incentivized decision-making paradigms, most commonly involving monetary rewards. In such tasks, individuals are presented with choices between smaller, immediately available rewards and larger rewards available after a specified delay. By systematically manipulating reward magnitudes and delay intervals, individual discounting preferences can be quantified.

Historically, DRD has been conceptualized as a facet of impulsivity, particularly as an indicator of impulsive decision-making (e.g., Ainslie, 1975; Green et al., 1996; Kirby et al., 1999; Mazur et al., 1987). However, this view has recently been subject to increasing debate. Strickland et al. (2021) argue that impulsivity does not meet the basic requirements of a construct, given its limited consistency with psychometric, clinical, and neuroscientific evidence, and should therefore be reconsidered. Instead, they propose replacing the umbrella construct of impulsivity with four core constructs—response inhibition, attention, risk sensitivity, and delayed consequence sensitivity—with DRD tasks considered relatively pure measures of delayed consequence sensitivity.

Bickel et al. (2012) suggested that temporal discounting is an underlying trans-disease process influencing various forms of maladaptive behaviors, such as addictive behaviors, problem gambling, and poor health practices, all of which are characterized by an inability to delay gratification and a preference for immediate rewards despite negative long-term consequences. DRD has been incorporated into the Research Domain Criteria (RDoC) framework within the Positive Valence Systems domain, under the construct of Reward Valuation and the subconstruct of Reward Delay, reflecting its role as a paradigm in reward-based decision-making processes across disorders (Insel et al., 2010; National Institute of Mental Health, 2016). DRD is strongly associated with problem gambling (Petry & Casarella, 1999; Ring et al., 2022) as well as with pathological gambling (Albein-Urios et al., 2014; Amlung et al., 2017; Dixon et al., 2003; Krmpotich et al., 2015; Michalczuk et al., 2011). It is therefore a well-established finding in cross-sectional studies that gamblers exhibit greater impulsivity in temporal decision making and discount future rewards more steeply (Albein-Urios et al., 2014; Amlung et al., 2017; Dixon et al., 2003; Krmpotich et al., 2015; Michalczuk et al., 2011; Petry & Casarella, 1999; Ring et al., 2022). The only longitudinal study known to us investigating this relationship described a bidirectional relationship between steep DRD and problem gambling in adolescence (Secades-Villa et al., 2016). As an underlying trans-disease process that plays a central role in both the etiology and maintenance of problem gambling, interventions targeting DRD should also be incorporated into therapy. Although DRD is considered a trait (Odum, 2011), it does appear to be malleable (Rung et al., 2019; Secades-Villa et al., 2014). Based on 92 studies, a meta-analysis by Rung et al. (2019) examined which interventions were most effective in changing DRD. Some interventions were suitable for one-time decisions (e.g., priming, framing), while others, such as episodic future thinking and learning-based approaches, were also able to reduce DRD in the long term.

Although only 20% of individuals with gambling problems seek professional help (Bijker et al., 2022) cognitive-behavioral therapies (CBT), including motivational interviewing lead to improvements in problem and pathological gambling (Gooding & Tarrier, 2009; Pallesen et al., 2005; Petry et al., 2017). Improvements in symptoms can also be achieved with web-based self-help interventions (Boumparis et al., 2023), which have the advantage of offering anonymous low-threshold treatment. However, elevated drop-out rates have been reported in both face-to-face therapy (Ladouceur et al., 2001) and web-based self-help interventions for problem gambling (Boumparis et al., 2023). Understanding the nature of dropout is an important research and clinical issue. DRD has been associated with drop-out in gambling disorder treatment (Mena-Moreno et al., 2022) and non-adherence to treatment in other diseases, such as breast cancer and diabetes (Lebeau et al., 2016; Vaughn et al., 2021). Given the limited evidence available, it is crucial to investigate the relationship between non-adherence and DRD, specifically in the context of problem gambling.

The goal of the current study is to extend the findings of the existing literature by analyzing longitudinal data from a large sample of a randomized controlled trial (Boumparis et al., 2023) with baseline and follow-up assessments at 8 weeks and 6 months to draw conclusions and inferences for a better understanding of the role of DRD. We aimed to explore the association of DRD with problem gambling and potential changes in DRD over the course of the original RCT. Additionally, we investigated whether changes in DRD mediate the relationship between the intervention and the reduction of gambling and other comorbid symptomatology, as well as the influence of DRD rates on participants’ adherence.

Four research questions were addressed in this secondary analysis. The following four hypotheses were formulated:

1. Steep DRD (i.e., higher *k* value) is associated with more pronounced symptomatology and greater severity of problem gambling.
2. Steep DRD is a predictor of low participant adherence.
3. DRD (*k* value) will decrease over the course of the study.
4. The effect of the Win Back Control intervention on the symptomatology and severity of problem gambling, as well as on the reduction of symptoms of psychopathological comorbidity (depression, anxiety, cigarettes smoked), is mediated by DRD.

## Methods

### Design

We conducted a secondary analysis of the Win Back Control data (Baumgartner et al., 2019; Boumparis et al., 2023). As part of this analysis, we excluded individuals who either did not complete the Monetary Choice Questionnaire (MCQ) (Kirby et al., 1999) or had consistency rates below 70% in the MCQ, as recommended by Gray et al. (2016). This led to the exclusion of 15 participants in total, 9 due to incomplete MCQ data and 6 due to low consistency rates. Consequently, the final sample consisted of 345 individuals, with 182 assigned to the intervention group and 163 to the active control group. Dropout rates were substantial, with only 19% of participants remaining at the 8-week follow up (*n* = 67) and 21% at the 6-month follow-up (*n* = 73).

The original study evaluated the web-based self-help intervention Win Back Control through a two-arm randomized controlled trial design. After completing the baseline assessments, participants were randomly assigned to either the active control group, which received the evidence-based self-help manual Becoming a Winner: Defeating Problem Gambling (Hodgins et al., 2000) or the Win Back Control intervention. Win Back Control is an automated, web-based self-help tool for problem gambling. The intervention comprises a gambling diary, a dashboard, and nine modules aimed at reducing gambling behavior and comorbid symptomatology, incorporating principles of motivational interviewing (Rollnick & Miller, 1995), self-control techniques, and cognitive-behavioral therapy (Meichenbaum, 1977). The modules are derived from previous web-based interventions for cannabis and alcohol use (Schaub et al., 2013, 2016) as well as the self-help manual ‘Becoming a Winner’ (Hodgins et al., 2000).

The MCQ was administered during the baseline assessment and at the 8-weeks and 6-month follow-up assessments.

The Ethics Committee of the Canton of Zurich approved the study on December 18, 2018 (BASEC No. 2018–01989), and it was registered in the ISRCTN registry (ISRCTN16339434).

A more detailed overview of the methodology can be found in the previously published studies (Baumgartner et al., 2019; Boumparis et al., 2023).

### Participants

Participants were recruited through the Win Back Control websites (winbackcontrol.ch, genuggespielt.at) between March 2019 and April 2021. The inclusion criteria were: (1) a minimum age of 18 years, (2) gambling at least once a week in the past 30 days, (3) a Problem Gambling Severity Index (PGSI) score above three, (4) internet access at least once a week and a valid email address, and (5) proficiency in either German or French. Exclusion criteria were: (1) participation in other psychosocial interventions for the treatment of problem gambling, (2) experiencing a psychotic or manic episode in the last 90 days, or (3) a prevalent severe substance use disorder. The latter was assessed with the Drug Abuse Screening Test (DAST-10) (Bohn et al., 1991) and the Alcohol Use Disorders Identification Test (AUDIT) (Saunders et al., 1993). Additionally, individuals with a suicidality level higher than minimal risk according to the P4 Suicidality Screener (Dube et al., 2010) were excluded. All participants who completed the 6-month follow-up assessment received a voucher worth 30 Swiss Francs (30 Euros) or the option to donate this amount to charity.

### Procedures

Participants registered online by providing their email address, phone number, and basic demographic details (age, gender, education level, and household income). After meeting the inclusion and exclusion criteria and giving informed consent, eligible participants were randomly assigned to either the intervention or the active control group. The allocation sequence was automatically generated by a computerized system using a 1:1 ratio.

### Measurements

DRD was operationalized using the MCQ (Kirby et al., 1999), which consists of 27 items, each requiring a choice between a smaller immediate reward (SIR) and a larger delayed reward (LDR). From these 27 items, a discounting rate can be calculated for each individual. An individual’s rate of delayed discounting is typically quantified as a hyperbolic function and can be determined using the following equation proposed by Mazur (1987) and later described by Gray et al. (2016).

$$\:V=A/\:(1+kD)$$

Equation (1) describes how the value (V) of a reward of a given amount (A) decreases as a function of the delay (D) until its receipt (Mazur et al., 1987). Higher *k* values indicate a stronger preference for immediate rewards, reflecting greater delayed discounting.

Problem gambling severity was assessed via the PGSI (Ferris et al., 2001). The PGSI consists of nine items, which can be categorized as gambling behavior or adverse consequences, each rated on a four-point scale. All items refer to the participants’ experiences during the past 12 months. Summing the scores of the individual items yields a total score ranging from 0 to 27, with a score of eight or higher indicating problem gambling. Severity of gambling symptoms was assessed via the Gambling Symptom Assessment Scale (G-SAS), a twelve-item self-report questionnaire designed to assess the severity of gambling symptoms and their changes during treatment (Kim et al., 2009). All G-SAS items refer to the past seven days and have a scoring range from 0 (no symptoms) to 4 (extreme symptoms). Total scores range from 0 to 48 and can be categorized as follows: mild (8–20), moderate (21–30), severe (31–40), and extreme (41–48). Depression was screened using the Patient Health Questionnaire-9 (PHQ-9) (Kroenke et al., 2001). The PHQ-9 consists of nine items, each corresponding to one of the nine DSM-IV criteria, and refers to the participant’s experiences during the past 14 days. Total scores range from 0 to 27, with the following severity categories: 5–9 indicating mild depression, 10–14 moderate depression, and 15–27 severe depression. Generalized anxiety symptoms were assessed using the Generalized Anxiety Disorder 7 (GAD-7) (Löwe et al., 2008), which consists of seven items and refers to the past 14 days. Each item is rated on a four-point Likert scale ranging from “not at all” to “nearly every day.” Total scores range from 0 to 21, with the following severity categories: 0–4 no anxiety, 5–9 indicating mild anxiety, 10–14 moderate anxiety, and 15–21 severe anxiety. To assess the use of cigarettes, we used the timeline followback (TLFB) method (Robinson et al., 2014). The PGSI, GSAS, MCQ, and TLFB for smoking were administered at all three timepoints, whereas the PHQ-9 and GAD-7 were only assessed at the 6-month follow-up, in line with the design of the primary study, which evaluated depressive and anxiety symptoms as secondary outcomes at this timepoint.

As participation in the study involved both the independent completion of online modules and the completion of various questionnaires at all measurement points, adherence was operationalized using two variables: the number of Win Back Control modules completed and a variable indicating the number of missing follow-up assessments out of the two possible for each individual.

### Statistical Analyses

The Monetary Choice Questionnaire was analyzed using the syntax provided by Gray et al. (2016) with R (Version 4.4.0; (R Core Team, 2021). To assess participants DRD rate, their individual *k* values were calculated according to Gray et al. (2016) using a hyperbolic function provided by Mazur (1987). A higher *k* value indicates a stronger degree of discounting by the individual (Gray et al., 2016; Kirby et al., 1999). An overall *k* value was subsequently computed by averaging the *k* values across the different magnitudes (Kaplan et al., 2016). This resulted in a single *k* value per individual for each measurement time point.

All data were analyzed according to the intention-to-treat (ITT) principle. Missing data for the ITT analyses were addressed using multiple imputation procedures with the R package mice (Version 4.1.2; (Buuren & Groothuis-Oudshoorn, 2011) for multivariate imputation by chained equations. Following the recommendation, 20 imputation sets were used (Buuren & Groothuis-Oudshoorn, 2011). To assess the robustness of the findings, a pre-specified complete case analysis (CCA) was conducted using the same model specifications but without robust standard errors. For comparability, the ITT analyses were also recomputed without robust standard errors. As these analyses served only as a methodological sensitivity check, detailed numerical results are not reported but are available upon request.

(H1) Due to the nested data structure, the relationship between the MCQ and the G-SAS and the PGSI, was modeled using separate linear mixed models (LMMs). Models were created using the R package lme4 (Version 1.1–35.4; (Bates et al., 2014). To account for heteroscedasticity, the models were also estimated with robust standard errors using the robustlmm package (version 3.3-1.3; (Koller, 2016). In total, we fitted and compared 4 models. Following the analysis strategy by Hox et al. (2017), we fitted (a) the null model in an initial step, and subsequently added (b) level-1 predictors, (c) random effects, and (d) level-2 predictors. We compared model fit via deviance tests.

(H2) Given the overdispersed count data, a negative binomial model was applied using the MASS package (version 7.3–60.2; (Venables & Ripley, 2002). The number of completed modules was used as the dependent variable, with the *k* value of the MCQ at baseline serving as the predictor. To test whether steep DRD at baseline predicts non-completion of the follow-up questionnaires, a multinomial logit model was estimated. The VGAM package (version 1.1–11; (Yee, 2015) was used for this analysis.

(H3) Due to heteroscedasticity, the robustlmm package was also used when testing the third hypothesis. The *k* value of the MCQ was designated as the dependent variable. Following again the analysis strategy by Hox et al. (2017), we fitted (a) the null model in an initial step, and subsequently added (b) level-1 predictors, (c) random effects, and (d) the level-2 predictor. Finally, a possible cross-level interaction between time and condition was tested, and model fit was compared via deviance tests.

(H4) Bootstrap mediation analyses were conducted using the mediation package (version 4.5.0; (Tingley et al., 2014). Five models were created, and change scores were computed for the dependent variables at the 8-week and 6-month follow-up. The dependent variables selected were those that were significantly reduced by the Win Back Control intervention in the Boumparis et al. (2023) study: PGSI scores, G-SAS scores, smoked cigarettes, PHQ-9 and GAD-7 scores. In each analysis, the Average Causal Mediation Effect (ACME) was estimated to assess the indirect effects of the intervention through the mediating variables. Additionally, the Average Direct Effect (ADE), the Total Effect, and the proportion mediated (prop. mediated) were calculated to quantify the portion of the total effect accounted for by the mediator.

All values are reported to two decimal places, except coefficients smaller than |0.01|, which are displayed with three decimals to avoid rounding to zero.

## Results

### Baselines Characteristics

Of the 345 participants, 239 were male (69.2%). The mean age was 33.43 years (SD = 10.37), and the majority resided in Germany (*n* = 188; 54.5%), followed by Switzerland, where 31.6% of the participants lived (*n* = 109). At baseline, participants had an average PGSI score of 16.70 (SD = 4.84), which falls in the problem gambling range of severity (score ≥ 8), a GSAS score of 32.35 (SD = 7.65) indicating severe gambling symptomatology (score ≥ 25), and a *k* value of 0.08 (SD = 0.08). The mean PHQ-9 score was 8.82 (SD = 4.60) indicating mild depressive symptoms (score 5–9), and the GAD-7 score was 9.90 (SD = 4.93), suggesting moderate anxiety symptoms (score 10–14). On average, participants smoked 85.64 cigarettes (SD = 100.10) in the seven days prior to baseline. Baseline characteristics are summarized in Table 1. Baseline characteristics were comparable across treatment groups, consistent with the findings reported by Boumparis et al. (2023). No significant differences were observed in demographic or clinical variables.

**Table 1:** Baseline participant data

Characteristics	Win Back Control *N* = 182	Self-help Manual *N* = 163
Gender, n		
Female	61 (33.5%)	45 (27.6%)
Male	121 (66.5%)	118 (72.4%)
Age, Mean (SD)	33.52 (10.70)	33.33 (10.02)
Country of residence		
Germany	97 (53.2%)	91 (55.8%)
Switzerland	61 (33.5%)	48 (29.4%)
Austria	20 (10.9%)	23 (14.1%)
Liechtenstein	1 (0.55%)	0 (0.00%)
Not specified	3 (1.65%)	1 (0.61%)
Education		
Primary school/General secondary school	30 (16.5%)	31 (19.0%)
Secondary school	59 (32.4%)	57 (35.0%)
Higher education entrance qualification	56 (30.8%)	31 (19.0%)
University/University of applied sciences	31 (17.0%)	35 (21.5%)
Special needs school	2 (1.09%)	1 (0.61%)
Not specified	4 (2.20%)	8 (4.91%)
Outcomes, mean (SD)		
PGSI	16.63 (4.97)	16.78 (4.70)
G-SAS	32.03 (8.19)	32.71 (7.01)
*K* value (MCQ)	0.09 (0.08)	0.08 (0.08)
ICR (MCQ)	0.67 (0.27)	0.66 (0.26)
PHQ9	8.76 (4.43)	8.89 (4.78)
GAD-7	9.89 (5.09)	9.91 (4.75)
Cigarettes smoked last 7 days	87.10 (114.23)	84.02 (84.32)

*Note*. *GAD-7* Generalized Anxiety Disorder-7, *G-SAS* Gambling Symptom Assessment Scale, *ICR* Inconsistency Rate, *MCQ* Monetary Choice Questionnaire, *PGSI* Problem Gambling Severity Index, *PHQ-9* Patient Health Questionnaire-9

## Main Outcomes

### (H1) Associations of DRD with Gambling Symptom Severity (G-SAS) and Problem Gambling Severity (PGSI)

As shown in Table 2, the model incorporating both time and the *k* value as predictors significantly predicted the G-SAS (time: *b* = −0.49, *p* < 0.001; *k* value: *b* = 13.90, *p* = 0.002) and the PGSI (time: *b* = −0.36, *p* < 0.001; *k* value: *b* = 9.39, *p* < 0.001). For the G-SAS, the model with a random slope for time provided the best fit, whereas for the PGSI, the best fit was achieved with a model that included only a random intercept. For the PGSI outcome, the interaction between time and the *k* value reached significance (*b* = − 0.49, *p* = 0.034). However, interaction terms did not improve model fit and were therefore not retained; the tables report final models only.

**Table 2:** LMM with robust standard errors

Best fitting model$\:{\:}^{a}$	G-SAS$\:{\:}^{b}$				PGSI$\:{\:}^{b}$				K value$\:{\:}^{c}$			
	b	SE b	95% CI	*p*	b	SE b	95% CI	*p*	b	SE b	95% CI	*p*
Intercept	29.42	0.57	28.30–30.54	**< 0.001**	12.87	0.36	12.16 − 13.58	**< 0.001**	0.06	0.00	0.06–0.07	**< 0.001**
Time	−0.49	0.03	−0.55 – −0.45	**< 0.001**	−0.36	0.02	−0.40 − −0.32	**< 0.001**	−0.001	0.000	−0.001–0.000	**< 0.001**
*K* value	13.90	4.41	5.19–24.41	**0.002**	9.39	2.55	4.39–14.39	**< 0.001**				

Note. All values are rounded to two decimal places, except coefficients smaller than |0.01|, which are displayed with three decimals for clarity. *LMM* Linear Mixed Model, *G-SAS* Gambling Symptom Assessment Scale, *PGSI* Problem Gambling Severity Index, *SE* Standard Error, *CI* Confidence Interval

^a^Time as random slope for the model with G-SAS as the criterion variable. The best model with PGSI as criterion variable contains no random effects

^b^Results from the first analysis testing H1

^c^Results from the third analysis testing H3

### (H2) DRD Adherence Prediction

As shown in Table 3, the negative binomial model (*b* = −1.50, *p* = 0.131) did not indicate a significant effect of baseline DRD on the number of completed modules of the Win Back Control intervention.

**Table 3:** Negative binomial model

Criterion Variable: Number of Completed WBC Modules				
Predictors	b	SE	95% CI	*p*
(Intercept)	0.99	0.11	0.77–1.22	**< 0.001**
*K* value at baseline	−1.50	0.99	−3.44–0.46	0.131

*Note*. *N *182, participants in the Win Back Control condition, *WBC* Win Back Control, *SE* Standard Error, *CI* Confidence Interval

Table 4 presents the results of testing the hypothesis that higher discounting rates predict non-participation at follow-up. The criterion variable in this multinomial logistic regression is the number of missed assessments (0, 1, or 2), with two missed assessments serving as the reference category. The coefficient of −3.89 suggests that an increase in the *k* value is associated with a decrease in the log-odds of having no missing measurement time points, compared to having two missing time points. However, this effect does not reach statistical significance at the 5% level (*p* = 0.078). Similarly, the coefficient of −0.71 indicates that an increase in the *k* value slightly reduces the log-odds of having one missing measurement time point, compared to two missing time points, but this effect is also not statistically significant (*p* = 0.717).

**Table 4:** Multinomial logit model

Criterion Variable: Missing assessment time point (0, 1, or 2; reference = 2)				
Predictors	b	SE	95% CI	*p*
(Intercept):1	−1.39	0.22	−1.81 – −0.96	**< 0.001**
(Intercept):2	−1.64	0.23	−2.09 – −1.19	**< 0.001**
*K* value at baseline:1	−3.89	2.21	−8.21–0.43	0.078
*K* value at baseline:2	−0.71	1.96	−4.56–3.14	0.717

*Note*. *N* 345, (Intercept), log-odds of 0 missing assessments vs. 2, (Intercept) 2, log-odds of 1 missing assessment vs. 2, k-value at baseline 1, effect of baseline discounting on log-odds of 0 missing assessments vs. 2, k-value at baseline 2, effect on log-odds of 1 missing assessment vs. 2. *SE* Standard Error, *CI* Confidence Interval

### (H3) DRD Temporal Change

The results of the third analysis are also presented in Table 2. The numerical variable time (measured in weeks) emerged as a significantly negative predictor of the *k* value (*b* = − 0.001, *p* < 0.001). The study condition was excluded from the final model as it did not reach statistical significance. Likewise, the interaction between the study condition and time was tested but not included in the model due to a lack of significance. Across the three assessment points, the aggregate *k* values declined from baseline (M = 0.08, SD = 0.08) to the 8-week follow-up (M = 0.07, SD = 0.08) and further to the 6-month follow-up (M = 0.06, SD = 0.08), illustrating an overall reduction in discounting rates over time.

### (H4) DRD Mediational Pathway

The bootstrap mediation analyses are presented in Tables 5, 6, 7 and 8. None of the examined ACME were found to be significant.

**Table 5:** Bootstrap mediation analysis: effect of the intervention group on changes in PGSI scores mediated by changes in the k value

	Change scores between baseline and 8-week follow-up			Change scores between baseline and 6-month follow-up		
	*estimate*	*95% CI*	*p*	*estimate*	*95% CI*	*P*
ACME	0.01	−0.08–0.10	0.884	−0.02	−0.14–0.09	0.767
ADE	1.73	0.44–3.04	**< 0.001**	2.16	0.89–3.38	**< 0.001**
Total Effect	1.74	0.44–3.06	**0.011**	2.14	0.87–3.38	**< 0.001**
Prop. Mediated	0.00	−0.07–0.08	0.882	−0.00	−0.09–0.05	0.767

*Note*. *ACME* Average Causal Mediation Effect, *ADE* Average Direct Effect, Prop. Mediated, Proportion Mediated, ACME/Total Effect, *PGSI* Problem Gambling Severity Index, *CI* Confidence Interval

**Table 6:** Bootstrap mediation analysis: effect of the intervention group on changes in G-SAS scores mediated by changes in the k value

	Change scores between baseline and 8-week follow-up			Change scores between baseline and 6-month follow-up		
	*estimate*	*95% CI*	*p*	*estimate*	*95% CI*	*P*
ACME	0.01	− 0.15–0.20	0.93	−0.05	−0.39–0.23	0.71
ADE	−0.53	− 2.90–1.86	0.65	4.19	1.71–6.65	**< 0.001**
Total Effect	−0.52	−2.89–1.87	0.66	4.14	1.68–6.60	**0.001**
Prop. Mediated	0.00	−0.59–0.70	0.99	−0.01	−0.13–0.06	0.71

*Note*. *ACME*, Average Causal Mediation Effect, *ADE* Average Direct Effect, Prop. Mediated, Proportion Mediated, ACME/Total Effect, *G-SAS* Gambling Symptom Assessment Scale, *CI* Confidence Interval

**Table 7:** Bootstrap mediation analysis: effect of the intervention group on changes in smoked cigarettes mediated by changes in the k value

	Change scores between baseline and 8-week follow-up			Change scores between baseline and 6-month follow-up		
	*estimate*	*95% CI*	*p*	*estimate*	*95% CI*	*P*
ACME	−0.13	−1.57–1.01	0.82	−0.33	−2.57–1.59	0.72
ADE	−9.74	−25.65–5.93	0.23	43.19	25.94–60.20	**< 0.001**
Total Effect	−9.86	−25.81–5.90	0.23	42.86	25.80–59.89	**< 0.001**
Prop. Mediated	0.00	−0.32–0.39	0.85	−0.00	−0.07–0.04	0.72

*Note*. *ACME* Average Causal Mediation Effect, *ADE* Average Direct Effect, Prop. Mediated, Proportion Mediated, ACME/Total Effect, *CI* Confidence Interval

**Table 8:** Bootstrap mediation analysis: effect of the intervention group on changes in PHQ-9 and GAD-7 scores mediated by changes in the k value

	Change score of the PHQ-9 between baseline and 6-month follow-up			Change scores of the GAD-7 between baseline and 6-month follow-up		
	*estimate*	*95% CI*	*p*	*estimate*	*95% CI*	*P*
ACME	−0.01	−0.13–0.08	0.77	−0.01	−0.09–0.07	0.88
ADE	2.50	1.42–3.57	**< 0.001**	3.86	2.74–5.03	**< 0.001**
Total Effect	2.49	1.41–3.54	**< 0.001**	3.86	2.72–5.03	**< 0.001**
Prop. Mediated	−0.00	−0.06–0.03	0.77	−0.00	−0.03–0.02	0.88

*Note*. *ACME* Average Causal Mediation Effect, *ADE* Average Direct Effect, Prop. Mediated, Proportion Mediated, ACME/Total Effect, *PHQ − 9* Patient Health Questionnaire-9, *GAD-7 *Generalized Anxiety Disorder-7, *CI* Confidence Interval

### Sensitivity Analysis

Results from the pre-specified CCA were highly consistent with the ITT findings, showing comparable effect directions and magnitudes, even when both analyses were computed without robust standard errors.

## Discussion

This study demonstrates that, as hypothesized, individuals who are more impulsive as indicated by steeper DRD exhibit significantly more severe gambling symptoms and a higher severity of problematic gambling behavior. Consequently, these results are consistent with those reported in the existing literature (Petry & Casarella, 1999; Ring et al., 2022). While the present study does not allow for causal conclusions, the findings highlight the potential value of considering DRD as a factor in the treatment of problematic gambling behavior. This conclusion is consistent with the results of Amlung et al. (2017), who, in their meta-analysis on various addictions, arrived at a similar conclusion. Based on Boumparis et al. (2023), and replicated in our analysis for hypothesis (1), both gambling symptom severity and comorbid psychopathology decreased over the course of the study. Notably, DRD did not moderate gambling symptom change over time; although higher DRD was associated with greater severity, trajectories were parallel across DRD levels, indicating comparable improvement irrespective of DRD.

Contrary to hypothesis (2), no association was found between DRD and the number of completed therapy modules, nor between DRD and participation at the measurement points. The relationship between DRD and adherence to gambling treatment, operationalized differently than merely through intervention dropout (Mena-Moreno et al., 2022), has not been examined prior to this work. This study is, therefore, the first to investigate the association of a non-dichotomous adherence variable in problematic gambling with DRD. One possible explanation for the absence of an association between DRD and the number of completed WBC modules is that the model included only baseline DRD values, whereas treatment adherence reflects behavior over several weeks. Because delay discounting has been shown to be sensitive to both trait and state influences (Odum, 2011; Wagner et al., 2022), restricting the analysis to baseline DRD values may not adequately capture its dynamic nature. Finally, as the analysis of completed modules was based solely on participants in the intervention group, the relatively small sample size and wide confidence interval indicate considerable uncertainty around the effect estimate, limiting the ability to draw firm conclusions about small effects.

Consistent with hypothesis (3), DRD significantly decreased across the measurement points. This study aligns with the meta-analysis by Rung et al. (2019), which demonstrated that temporal discounting is malleable. The study thus yielded results similar to those of Secades-Villa et al. (2014), who found that adolescent smokers exhibited lower DRD rates 12 months after treatment compared to baseline. Consistent with these findings, Landes et al. (2012) reported comparable reductions in DRD following opioid treatment. To our knowledge, however, no study to date has investigated longitudinal changes in DRD and problem gambling across multiple assessment points in the context of gambling treatment. In the present study, the change in DRD did not differ between study conditions: in both the Win Back Control condition and the active control group, DRD decreased over time. It is conceivable that both treatment conditions contributed to a reduction in DRD.

In line with this, hypothesis (4)—which posited that changes in DRD mediate the relationship between the Win Back Control intervention and reductions in gambling symptom severity and psychopathological comorbidity—was not supported, as no significant indirect effect was detected. Several explanations may account for this finding. First, both treatment conditions led to comparable reductions in DRD and gambling symptoms, resulting in limited variance in the treatment factor and thus constraining the possibility of detecting a mediation effect. Second, DRD and symptom improvement may reflect distinct but parallel processes rather than a causal sequence. Finally, without a passive control condition, it cannot be ruled out that the observed reductions in DRD reflect, at least in part, nonspecific factors such as repeated assessment or study engagement rather than treatment-specific mechanisms. Future studies should therefore include a wait-list control group to disentangle these effects and more precisely evaluate whether changes in DRD mediate treatment-related improvements in gambling symptoms.

Given these findings, it remains unclear whether the reduction in DRD contributed to symptom improvement, whether symptom improvement led to lower discounting, whether both processes mutually influenced each other over time, or whether both were affected by a third variable. Clarifying the directionality of this relationship is an important task for future research, especially with regard to validating Bickel’s etiological model of DRD as a transdiagnostic process in the context of gambling addiction. Moreover, as DRD has been incorporated into the RDoC Matrix as a transdiagnostic construct (Insel et al., 2010; Levitt et al., 2022; National Institute of Mental Health, 2016), clarifying its role in the development and maintenance of such behaviors becomes even more critical. Lempert et al. (2019) raised the crucial question of whether interventions that alter DRD can lead to clinically significant improvements in health-related behavior. Episodic future thinking represents one such intervention (Atance & O’Neill, 2001; Rung et al., 2019); however, whether it can reduce gambling behavior through changes in DRD had not yet been investigated at the time of the present study.

### Limitations

The study, being a secondary analysis not planned in prior, had a few noteworthy limitations. Methodologically, it is a limitation that no wait list control group was available. However, to make statements about the trajectory of DRD and the reasons for the changes, a passive control group would be necessary. Boumparis et al. (2023) report a substantial dropout rate of 76%, raising validity concerns. We addressed this through multiple imputation, which reduces bias even with high attrition rates (Madley-Dowd et al., 2019) and outperforms listwise deletion under both missing-at-random (MAR) and missing-not-at-random (MNAR) conditions (van Ginkel et al., 2020). However, given the attrition magnitude and the likelihood of MNAR mechanisms, some bias may persist despite these precautions. Findings should be interpreted with this constraint in mind. Another limitation is the data collection, which is entirely based on self-report. Although there is evidence that self-reports on gambling behavior are a valid and reliable method for collecting data on individuals with gambling problems (Hodgins & Makarchuk, 2003).

Regarding generalizability, the limitations arise from the inclusion and exclusion criteria used in the original study Boumparis et al. (2023). Specifically, individuals who suffered from severe substance use disorders, experienced psychosis or manic episodes, or exhibited suicidal risk higher than “minimal” were excluded. This significantly reduces the generalizability of the study, as comorbidities are widespread among individuals with gambling problems (Allami et al., 2021; Buth et al., 2017). Furthermore, these restrictions reduce variance; for example, the PHQ-9 includes an item on suicidality. When variance in key predictors is restricted, this can lead to an underestimation of relationships due to range restriction.

Finally, it should be noted that neither intervention arm involved face-to-face contact, which may limit comparability to in-person interventions. Future studies could examine whether similar effects emerge in blended or fully in-person formats.

## Conclusions

In this secondary analysis of data from a two-arm RCT with three measurement time points, we aimed to examine the relationship between DRD and problem gambling, explore temporal changes in DRD, and assess its predictive value for adherence. The findings of the present study indicate a positive association between DRD, gambling symptoms, and problem gambling severity. DRD was not found to be a significant predictor of adherence. However, DRD was found to be decreasing in both the intervention and active control groups alongside reductions in the severity of symptomatology and gambling severity. The hypothesis that DRD mediates the effects of the online intervention on symptomatology and problem gambling severity could not be confirmed. Given its association with problem gambling and its modifiability, future research should investigate the directionality of this relationship through further RCTs. Specifically, it should be examined whether targeting DRD—through approaches like episodic future thinking, which has the potential to reduce DRD over the long term—can improve clinical outcomes, possibly making it a key component of future gambling treatments.

## Data Availability

The datasets generated and analyzed during the current study are not publicly available due to participant privacy and GDPR considerations. However, anonymized or aggregated data may be made available from the corresponding author upon reasonable, written request. Such requests will be subject to a review process, including an assessment of the proposed research question's alignment with ethical guidelines, data protection regulations, and the terms of participant consent.
